# FRACTURE-DISLOCATIONS OF THE ELBOW: CAN THEY INFLUENCE THE PATTERN FRACTURE OF RADIAL HEAD?

**DOI:** 10.1590/1413-785220243202e278639

**Published:** 2024-06-24

**Authors:** Thiago Augusto da Silva, Alexandre Martins Malaquias, Marcio Alves Cruz, Fernando Kenji Kikuta, Guilherme Grisi Mouraria, Maurício Etchebehere

**Affiliations:** 1 Universidade Estadual de Campinas (UNICAMP), Campinas, SP, Brazil.

**Keywords:** Radial Head and Neck Fractures, Elbow Fractures, Fracture Dislocation, Fraturas da Cabeça e do Colo do Rádio, Fraturas do cotovelo, Fratura-Luxação

## Abstract

**Introduction:**

Radial head fractures are consistently part of a terrible triad of the elbow and can occur in association with Monteggia fracture-dislocations, transolecranon fractures, and their variations. Understanding the degree of comminution of the radial head fracture and the location of fragments determines the course of action to be taken.

**Objectives:**

To correlate fracture-dislocations with the pattern of radial head fracture (number of fragments) and involvement in the proximal radioulnar region.

**Materials and Methods:**

A retrospective study (level II) of patients undergoing surgery for radial head fractures associated with fracture-dislocations. Patients had radiographs in anteroposterior and lateral views, as well as tomography. The number of radial head fracture fragments and the presence of fractures in the proximal radioulnar region were correlated with the type of fracture-dislocation and demographic variables.

**Conclusion:**

Elbow fracture-dislocation types could not predict the number of fragments and the location of radial head fractures. However, most injuries presented three or more fragments in the radial head, and many had involvement of the proximal radioulnar region, suggesting high-energy trauma. ***Level of Evidence II; Retrospective Study.***

## RESUMO

**Introdução:**

As fraturas da cabeça do rádio estão sempre presentes em uma tríade terrível do cotovelo e podem ocorrer associadas a uma fratura-luxação de Monteggia, fratura transolecraniana e suas variações. Conhecer o grau de cominuição da fratura da cabeça do rádio e a localização dos fragmentos determinam a conduta a ser tomada.

**Objetivos:**

Correlacionar as fraturas-luxações com o padrão da fratura da cabeça do rádio (número de fragmentos) e o acometimento na região radioulnar proximal.

**Material e Métodos:**

Estudo retrospectivo (nível II) de pacientes submetidos a cirurgia devido fraturas de cabeça de rádio associadas às fraturas-luxações. Os pacientes possuíam radiografia nas incidências anteroposterior e perfil e tomografia. O número de fragmentos da fratura da cabeça do rádio e a presença de fratura na região radioulnar proximal foram correlacionadas com o tipo de fratura-luxação e as varáveis demográficas.

**Conclusão:**

Os tipos de fratura-luxação do cotovelo não foram capazes de predizer o número de fragmentos e a localização da fratura da cabeça do rádio. Entretanto, a maioria das lesões apresentaram três ou mais fragmentos na cabeça do rádio e muitos apresentaram acometimento da região da radioulnar proximal sugerindo traumas de alta energia. ***Nível de Evidência II; Estudo Retrospectivo.***

## INTRODUCTION

Fractures of the radial head are common and account for one-third of elbow fractures, often caused by falls with the elbow in a pronated and partially flexed position, mainly affecting patients between 20 and 60 years of age .^1,2^ Radial head fractures are always part of a terrible triad of the elbow and can occur in association with a Monteggia fracture-dislocation, transolecranon fracture, and their variations.^3^ These injuries are related to high-energy traumas.^4^ One of the most commonly used classifications for radial head fractures is the Mason classification modified by Broberg and Morrey, defined as follows: type 1 - marginal fractures without displacement; type 2 - fragments involving at least 30% of the articular surface with more than 2mm displacement; type 3 - comminuted fracture involving the entire head, and type 4 when the radial head fracture is associated with elbow dislocation.^3^ Complementary exams such as X-rays and computed tomography assist in confirming the diagnosis and studying the patterns of radial head fractures. The correlation of the trauma mechanism (direction of dislocation) and the types of elbow fracture-dislocation with the pattern of radial head fracture can aid in therapeutic decision-making.^4^ The management of radial head fractures in elbow dislocations is complex and generally requires surgical treatment.^3^ Treating radial head fractures is crucial to achieve elbow stability after fracture-dislocation.^5^ Knowing details of the Radial Head fracture, such as involvement of the radioulnar joint, number of fragments, and direction of the main fragment, is essential in making surgical decisions. Fractures with three or more fragments and radioulnar involvement are prone to be treated with arthroplasty to achieve a stable and pain-free elbow. On the other hand, fractures with fewer than two fragments and no radioulnar involvement can be reconstructed with osteosynthesis with a good outcome.^6^ Thus, knowledge of the degree of comminution of the radial head fracture and the location of the fragments determines the course of action. There are few studies in the literature that correlate the type of elbow fracture-dislocation with radial head fracture patterns.

The primary objective of this study was to correlate the various types of fracture-dislocations with the pattern of radial head fracture (number of fragments).

The secondary objective was to correlate the presence of radial head fracture fragments in the proximal radioulnar region with the various types of elbow fracture-dislocation.

## MATERIALS AND METHODS

A retrospective study was conducted by reviewing medical records at a referral hospital between the years 2018 and 2023.

Inclusion criteria were as follows: patients undergoing surgical treatment for radial head fractures associated with Terrible Triad, Monteggia fracture, or transolecranon fracture. All patients should have had preoperative radiographs in anteroposterior and lateral views as well as preoperative tomography.

Patients who did not sign the informed consent form (ICF) or those with inadequate medical records/imaging for evaluation were excluded from the study.

**Figure 1:**
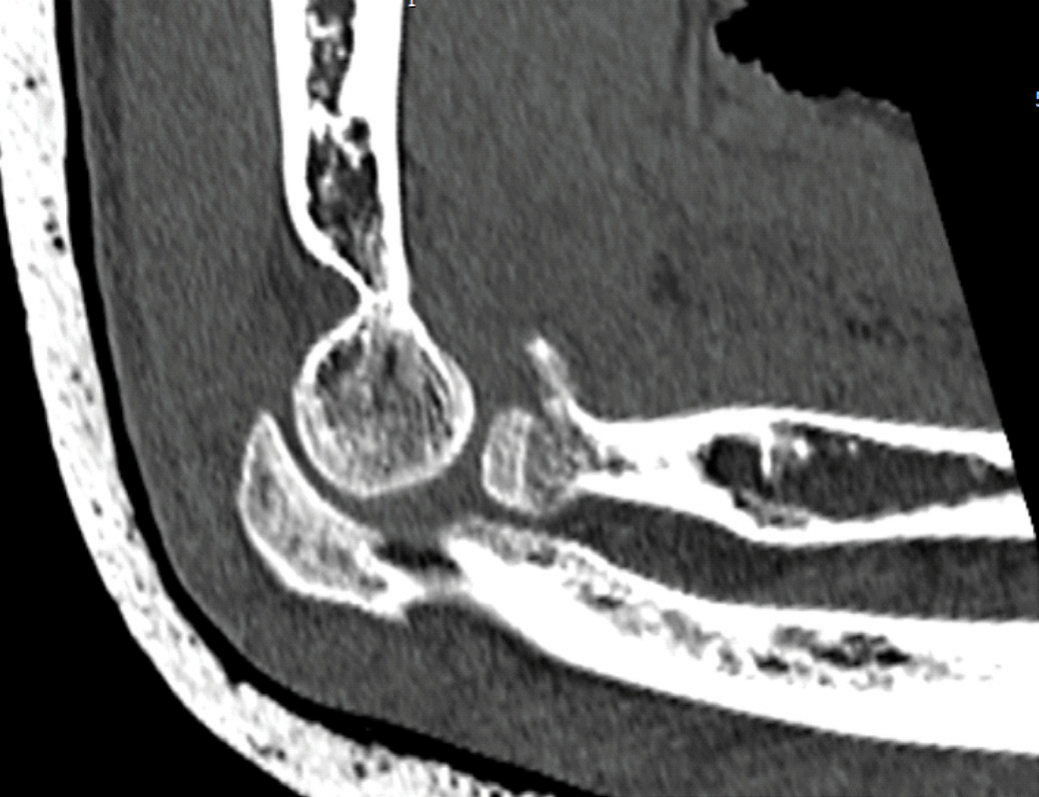
Method for considering a fragment in the radial head.

**Figure 2:**
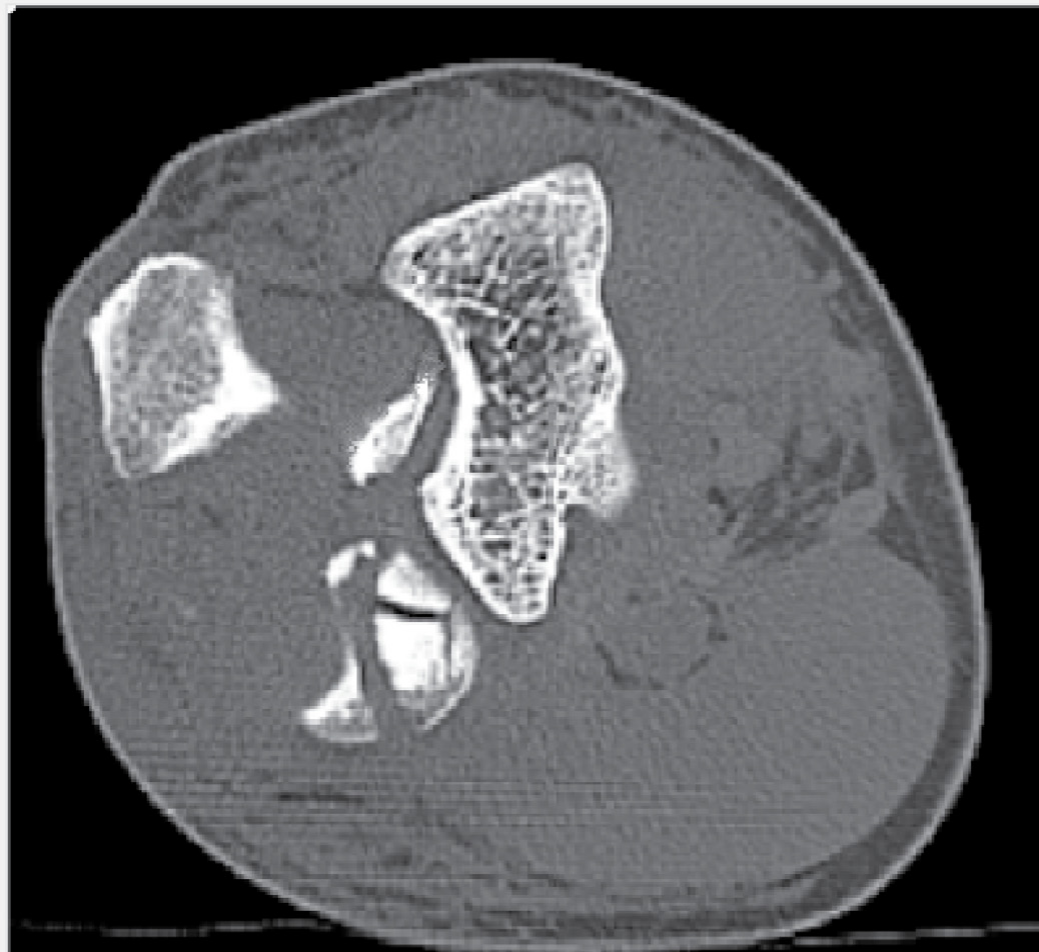
Fracture affecting the proximal radioulnar region.

Demographic data such as gender, age, and the affected side were analyzed. Radiological assessment was performed using the SynapseR program. The number of fragments and whether the fracture affected the proximal radioulnar region were analyzed. A fragment was considered present when there was a displacement greater than 2 mm, as calculated in the tomography examination (Figure 1). It was considered that the fracture affected the radioulnar joint when the radial head fragment was located in the area of the greater sigmoid notch of the ulna, as analyzed in the axial cut of the tomography (Figure 2). The direction of elbow dislocation was assessed in the lateral radiograph and the sagittal cut of the tomography.

The number of fragments in the radial head fracture and the presence of a fracture in the proximal radioulnar region were correlated with the type of fracture-dislocation and demographic variables. Categorical variables were tested using the chi-square test or Fisher's exact test. Non-categorical variables were tested using the Kolmogorov-Smirnov test. Therefore, both unpaired t-tests (parametric variables) and Mann-Whitney tests (non-parametric) were used in the study of these variables. All analyses were conducted using PASW Statistics 18.0 software (SPSS Inc., Chicago, USA), with a significance level of 5% (P < 0.05). The study was approved by the local ethics committee under number 66748122.6.0000.5404.

## RESULTS

Initially, 113 medical records were evaluated, but only 59 met all the inclusion criteria. The mean age was 43.8 ± 15 years (ranging from 21 to 80 years). There was a higher prevalence of males (64%). There was no difference in terms of the affected side of the body, with 50.8% on the right side and 49.2% on the left side. The most frequent injury was the terrible triad of the elbow (72.9%), followed by Monteggia fracture-dislocation (22%). Demographic data are described in Table 1.

**Table 1:** Demographic data.

Variable	Value
**Age [Average (± SD)] (years)**	43,83 ± 15,21
**Ses[n° (%)]**	
Man	38 (64,4%)
Woman	21 (35,6%)
**Type of injury[n° (%)]**	
Terrible elbow triad	43 (72,9%)
Monteggia fracture-dislocation	13 (22%)
Transolecranial fracture	3 (5,1%)
**Affected side[n° (%)]**	
Right	30 (50,8%)
Left	49,2%)

Regarding the number of fragments in the radial head fracture, it was observed that no patient had only one fragment (median of 3), as presented in Table 2.

**Table 2:** Number of radial head fracture fragments.

Number of fragments	Value N (%)
2	14 (23,7%)
3	27 (45,8%)
4	13 (22%)
5	4 (6,8%)

N = number; % = porcentage.

The type of fracture-dislocation (p=0.94) and the direction of elbow dislocation (p=0.71) did not influence the number of fragments in the radial head. In 17 patients, the radial head fracture affected the region of the proximal radioulnar joint. Despite the majority of patients having two or more fragments, the number of fragments did not influence the frequency of fractures affecting the radioulnar proximal region (p=0.80). Furthermore, the type of fracture-dislocation and the direction of dislocation did not influence the presence of a fracture in this region (Table 3).

**Table 3:** Incidence of fracture in the proximal radioulnar region compared with type of fracture, dislocation and direction of dislocation.

Type of injury	Fracture affecting the radioulnar region	Fracture not affecting the radioulnar region	Value of p^(a)^
**Classification**			
Terrible elbow triad (44)	10	34	0,077
Monteggia (12)	5	7	0,271
Transolecranial (3)	2	1	0,137
**Dislocation direction**			
Posterior (45)	13	32	0,268
Anterior (3)	2	1	0,137
Lateral (11)	2	9	0,388

N = total number; (a) Fischer's exact test.

## DISCUSSION

Fractures of the radial head are often accompanied by other elbow injuries, which can be fractures and/or dislocations. Haasters et al.^7^ describe that radial head fractures are associated with concomitant injuries in the elbow joint, which may be underdiagnosed in radiological examinations.

In this study, we investigated a correlation between the number of fragments in radial head fractures, the presence of an articular fragment (radioulnar joint), and the direction of dislocation, as these are important prognostic factors and surgical planning considerations.^7^ A 2009 study by Rinner (3) involving 296 patients with radial head fractures found associated injuries in 49% of cases, with the most common being the terrible triad of the elbow (19.9%), followed by posterior olecranon fractures-dislocations at 13.9%, and Monteggia lesions at 4.4%. Our results corroborate the literature data. We found only 3 cases (5.1%) of concomitant radial head fracture with transolecranon fracture, indicating it to be an infrequent association. In a study by Ditsios et al.^8^ involving 15 cases over 5 years of study, it was considered a rare association by the authors.

The average age of patients was 42.9 ± 10.9 years, and 64% were male. An epidemiological study conducted by Kodde et al.^6^ Showed similar data with an average age ranging from 44 to 47.9 years and gender ratios ranging from 1:1, 2:3, and 3:2 (male-female). Female patients are significantly older compared to male patients. The peak incidence in men occurs between 30 and 40 years, while in women, it is between 50 and 60 years. Therefore, the incidence peak is bimodal: young male patients and older female patients.^6^ Elbow fractures-dislocations, especially the terrible triad, often involve significant comminution of the radial head. Our results are consistent with the literature, as 74.6% of cases had 3 or more fragments. This result is similar to studies by Gonçalves et al.^9^ and Miyazaki et al.^10^

Understanding the specific characteristics of elbow fracture-dislocations is important because they influence treatment and prognosis. Gonzalez et al.^11^ Compared complication profiles and outcomes in patients associated with these two distinct patterns over a 12-year period. The authors evaluated 105 patients, 58 with Monteggia injuries and 47 with terrible triad injuries, and identified elbow stiffness as the main complication. Elbow contractures requiring surgical release were more commonly associated with terrible triad injuries.^11^

Hockmann et al.^12^ Emphasized that Monteggia-type fractures are complex injuries with high rates of complications, sequelae, and functional limitations. One possible cause of reduced range of motion may be related to fractures affecting the proximal radioulnar joint, which were not described in previous studies. We observed that 27% of cases had fractures in this region. Therefore, even if a radial head arthroplasty is performed, potential chondral damage to the ulna may be established, leading to functional limitations and/or pain. This study has some limitations. The first is related to the small number of patients, although these are relatively rare injuries compared to isolated radial head fractures. Another bias is related to determining the location of the fracture and the possible involvement of the proximal radioulnar region, as this location is influenced by forearm pronation and supination. Despite the limitations of this study, the proposal to analyze the characteristics of radial head fractures and correlate them with the types of elbow fracture-dislocations was important. Most studies do not mention the number of fragments and whether they involve the radioulnar joint.

## CONCLUSION

The types of elbow fracture-dislocations were not able to predict the number of fragments and the location of the radial head fracture. However, the majority of injuries showed three or more fragments in the radial head, and many of them involved the proximal radioulnar region, suggesting an association with high-energy trauma.
